# G-protein coupled receptors of the renin-angiotensin system: new targets against breast cancer?

**DOI:** 10.3389/fphar.2015.00024

**Published:** 2015-02-17

**Authors:** Sylvie Rodrigues-Ferreira, Clara Nahmias

**Affiliations:** Inserm U981, Institut Gustave RoussyVillejuif, France

**Keywords:** GPCR, Angiotensin, ARBs, Mas, breast cancer, therapy

## Abstract

G-protein coupled receptors (GPCRs) constitute the largest family of membrane receptors, with high potential for drug discovery. These receptors can be activated by a panel of different ligands including ions, hormones, small molecules, and vasoactive peptides. Among those, angiotensins [angiotensin II (AngII) and angiotensin 1–7] are the major biologically active products of the classical and alternative renin-angiotensin system (RAS). These peptides bind and activate three different subtypes of GPCRs, namely AT1, AT2, and Mas receptors, to regulate cardiovascular functions. Over the past decade, the contribution of several RAS components in tumorigenesis has emerged as a novel important concept, AngII being considered as harmful and Ang1–7 as protective against cancer. Development of selective ligands targeting each RAS receptor may provide novel and efficient targeted therapeutic strategies against cancer. In this review, we focus on breast cancer to summarize current knowledge on angiotensin receptors (AT1, AT2, and Mas), and discuss the potential use of angiotensin receptor agonists and antagonists in clinics.

The RAS is an endocrine system that plays a central role in cardiovascular and renal physiology through regulation of blood pressure and sodium balance. Deregulated RAS may contribute to pathogenesis such as atherosclerosis, ischemic disease, hypertension, and heart failure. The physiological effects of the RAS are mediated by bioactive angiotensin peptides generated by enzymatic cascades and released in the systemic circulation as well as locally in various tissues (Crowley and Coffman, 2012). The “classical RAS” produces the AngII octapeptide through cleavage of inactive AngI by angiotensin converting enzyme (ACE), whereas the “alternative RAS axis” (Santos et al., 2013) produces Ang1–7 by subsequent cleavage of AngII by the ACE homolog ACE2 (**Figure 1**). All Angiotensin peptides bind to GPCRs, namely AT1, and AT2 receptors for AngII and Mas receptor for Ang(1–7), but activate distinct signaling pathways leading to different and often opposite cellular effects (**Figure 1**). It is generally accepted that the classical ACE/AngII/AT1 axis promotes most of the RAS actions on cardiovascular, renal, and cerebral functions, and that these effects are counteracted by activation of the AT2 receptor (Porrello et al., 2009; McCarthy et al., 2013) and by the “protective” ACE2/Ang(1–7)/Mas axis (Santos et al., 2013).

**FIGURE 1:**
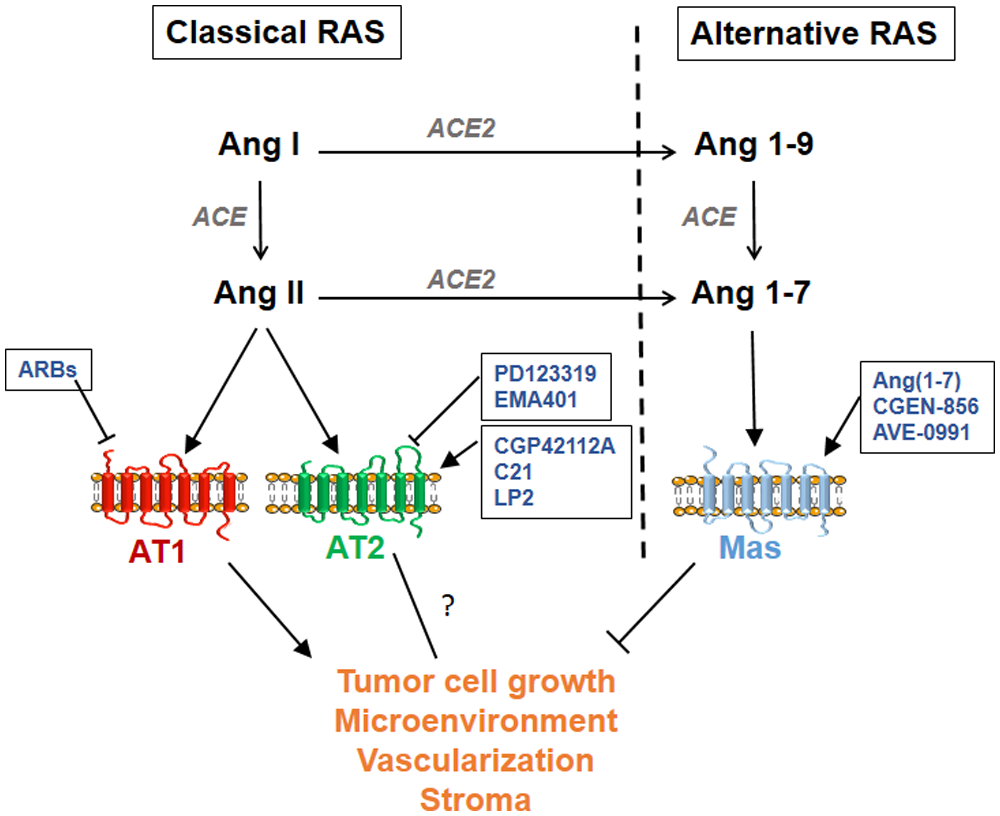
**Schematic representation of the classical and alternative renin-angiotensin system (RAS) pathways.** Available agonists and antagonists are indicated for each angiotensin G-protein coupled receptor (GPCR): AT1, AT2, and Mas. Available ARBs (AT1 receptor blockers) are Losartan, Candesartan, Valsartan, Irbesartan, Telmisartan, Eprosartan, Olmesartan, and Azilsartan.

Beyond cardiovascular functions, several studies have pointed out a role for RAS components in solid tumors including breast tumors (reviewed in George et al., 2010). Breast cancer is the major cause of death by cancer in women worldwide and the occurrence of distant metastasis is a critical event that limits patients survival. While targeted molecular therapies have considerably improved the management of primary breast tumors, these remain poorly effective for the treatment of metastases. The identification of molecular agents that may contribute to breast cancer progression is therefore essential for future development of new therapeutic strategies. Targeting GPCRs is of particular interest because of the availability of specific agonists and antagonists of the receptors. In addition, their cell surface location makes them suitable for blockade by humanized antibodies, a strategy successfully developed for HER2+ breast tumors with trastuzumab (Herceptin®). Of interest, most components of the RAS are locally expressed in breast tumors and microenvironment (macrophages and vascular cells; Inwang et al., 1997; De Paepe et al., 2001; Tahmasebi et al., 2006; Herr et al., 2008; George et al., 2010) pointing out angiotensin receptors as potential targets in breast cancer.

In this review, we summarize current knowledge on angiotensin receptors (AT1, AT2, and Mas) in breast cancer, and discuss the potential use of angiotensin receptor agonists and antagonists in malignancy.

## ANGIOTENSIN RECEPTORS IN CANCER

G-protein coupled receptors (GPCRs) of the renin-angiotensin system (RAS) system are activated by angiotensin peptides which are produced by angiotensin converting enzyme (ACE). ACE inhibitors (ACEi) have been assessed in cancer to characterize angiotensin functions in tumor growth, angiogenesis, and metastasis, and were shown to inhibit cell proliferation and tumor progression in several cancer models (reviewed in Rosenthal and Gavras, 2009). However, ACEi prevent production of both angiotensin II (AngII) and Ang1–7, thus preventing activation of all angiotensin receptors. Targeting specific angiotensin receptors using selective agonists or antagonists is warranted in order to validate their use to block tumor progression.

### AT1 RECEPTOR

The AT1 receptor is ubiquitously expressed in human tissues and is responsible for most of the AngII actions. AT1 activation by AngII triggers a large number of intracellular effectors leading to modulation of various cell processes, including proliferation, migration, angiogenesis, and inflammation, which are closely associated with tumor progression (Deshayes and Nahmias, 2005). Angiotensin receptor blockers (ARBs), such as losartan, candesartan, or valsartan, have been assayed both in cancer cells and in mouse experimental models to characterize AngII/AT1 functions in tumor growth, angiogenesis, and metastasis (George et al., 2010). Evidence for a role of AT1 receptor on cancer cell metastasis came from *in vivo* studies of lung models of metastasis. After injection of cancer cells into the tail vein of mice, oral administration of candesartan led to a strong reduction of lung metastasis (Miyajima et al., 2002). However, in this study it was not clear whether ARBs act on tumor cells or on the stromal microenvironment. The role of AT1 in the tumor microenvironment has been investigated by comparing the growth and vascularization of tumors injected subcutaneously into wild type (WT) or AT1 knockout mice (Egami et al., 2003; Fujita et al., 2005; Imai et al., 2007). Tumor growth and vascularization were strongly reduced in AT1 null mice indicating that the AT1 of host cells contributes to both tumor growth and angiogenesis. Of interest, AT1-dependent tumor growth involves an increase in VEGF synthesis, a well-known angiogenic factor (Fujita et al., 2005). Furthermore, AT1 is highly expressed in the stromal tissue surrounding the tumors, in particular in tumor-associated macrophages (TAMs). Macrophage infiltration, as well as levels of TAMs-released VEGF, were strongly reduced in AT1 null mice, supporting the hypothesis that host AT1 might also participate in inflammation-related tumor angiogenesis to maintain tumor growth (Egami et al., 2003; Fujita et al., 2005). In glial tumor patients, AT1 expression was associated with higher proliferation and vascular density and with reduced survival, indicating that AT1-expressing tumors are of poor prognosis (Arrieta et al., 2008).

### AT2 RECEPTOR

Angiotensin II also binds the AT2 receptor subtype but less is known about the functional consequence of AT2 receptor activation in cancer. *In vitro* studies indicate that over expression of AT2 reduces growth of lung adenocarcinomas cells (Pickel et al., 2010). In agreement, exogenous administration of AT2 receptor by nanoparticles was found to significantly attenuate lung cancer growth in an orthotopic model of syngeneic tumor grafts (Kawabata et al., 2012). AT2 receptor activation using the agonist CGP42112A reduced colorectal liver metastasis (Ager et al., 2010), suggesting that AT2 activation might provide a novel strategy to inhibit tumor growth. Of interest, pancreatic cancer cells subcutaneously injected in AT2 knockout mice grew significantly faster than in WT mice, indicating that AT2 receptors present in the tumor microenvironment may prevent cancer progression (Doi et al., 2010). However, in some other studies, the development of chemically induced sarcoma was delayed in AT2 knockout mice, and AT2 blockade by AT2 antagonist PD123319 significantly reduced lung carcinomas xenografts growth (Clere et al., 2010). Thus, further studies are needed to elucidate AT2 functions in cancer.

Studies on AT2 receptor signaling allowed the identification of several AT2 interacting partners that are related to cancer (Rodrigues-Ferreira et al., 2015). Among them, intracellular proteins of the ATIP family are encoded by candidate tumor suppressor gene *MTUS1*. *MTUS1* was shown to be down regulated in several solid tumors, including from pancreas (Seibold et al., 2003), ovary (Pils et al., 2005), head-and-neck (Ye et al., 2007; Ding et al., 2012), colon (Zuern et al., 2010), bladder (Xiao et al., 2012), and breast (Rodrigues-Ferreira et al., 2009), and ATIPs have been shown to display tumor suppressor effects (Seibold et al., 2003; Rodrigues-Ferreira et al., 2009). Investigating the functional relationship between AT2 and ATIPs might bring more clues toward understanding the effects of AT2 in cancer.

### MAS RECEPTOR

Angiotensins 1–7, the cleavage product of AngII by ACE2, belongs to the alternative RAS pathway and has protective effects on cardiovascular functions (Santos et al., 2013). Ang1–7 is an anti-proliferative and anti-angiogenic molecule that mediates its effects by binding to a unique GPCR, Mas (Santos et al., 2003; Passos-Silva et al., 2013).

The anti-proliferative and anti-angiogenic effects of the Ang1–7/Mas axis in cancer have been evaluated. *In vitro* studies on lung cancer cells confirmed the anti-proliferative function of Ang1–7 through Mas receptor (Gallagher and Tallant, 2004). *In vivo* studies further indicated that administration of Ang1–7 reduces lung and prostate tumor xenografts (Gallagher and Tallant, 2004; Menon et al., 2007; Krishnan et al., 2013a), as well as prostate cancer metastasis (Krishnan et al., 2013b). Of interest, Ang1–7 effect on tumor growth was associated with a strong effect on tumor microenvironment. Administration of Ang1–7 has been shown to act on endothelial cells to inhibit angiogenesis (Machado et al., 2001; Soto-Pantoja et al., 2009) but also on cancer-associated fibroblasts and osteoclasts to reduce breast cancer fibrosis and metastatic prostate tumor osteoclastogenesis, respectively (Cook et al., 2010; Krishnan et al., 2013b). All *in vitro* effects of Ang1–7 are inhibited by the Mas receptor antagonist A779 (Machado et al., 2001), suggesting that Mas receptor mediates all protective functions of Ang1–7.

## ANGIOTENSIN RECEPTORS IN BREAST CANCER

In breast tumors, most components of the RAS are expressed (George et al., 2010), suggesting that AngII may be locally produced. Studies from our group aimed at investigating the effect of AngII on breast cancer progression. To this end, metastatic breast cancer cells (D3H2LN) were exposed to AngII *in vitro* prior to injection into the blood flow of immunodeficient mice (Rodrigues-Ferreira et al., 2012a). Our studies revealed for the first time that AngII can directly act on breast cancer cells to promote cancer cell invasion and metastasis (Rodrigues-Ferreira et al., 2012a). Two major pathways activated by AngII were identified, that were related to cell proliferation and migration/invasion, respectively. These data highlight the importance of defining the role of angiotensin receptors in breast cancer as a first step toward blocking AngII production using ACEi or specifically targeting RAS GPCRs.

### AT1

AT1 has been shown to be expressed both at mRNA and protein levels in normal and malignant breast tissues (De Paepe et al., 2001; Tahmasebi et al., 2006; Herr et al., 2008; **Table 1**). Functional studies indicated that over expression of AT1 in breast cancer cells promotes cell invasion *in vitro* and tumor growth *in vivo* in absence of any stimulation by the AT1 agonist AngII (Rhodes et al., 2009). All these effects were dose-dependently inhibited by the AT1 antagonist losartan confirming the specific role of AT1. Blockade of AT1 by ARBs, such as losartan or candesartan, was shown to inhibit breast cancer cell proliferation *in vitro* and breast tumor xenograft growth in mice (Chen et al., 2013), suggesting that AT1 may be an interesting target against breast cancer. Interestingly, *in vitro* studies demonstrated that AT1 levels are increased in tamoxifen-resistant MCF7 cells as compared to sensitive counterparts, and that tamoxifen sensitivity was restored by blockade of AT1 by losartan (Namazi et al., 2014a,b).

**Table 1:** Renin-angiotensin system (RAS) G-protein coupled receptors (GPCRs) in breast cancer.

Receptor	Detection method	*In vivo* model	Observation	Reference
AT1	IHC/ISH		Increased in benign and malignant tumors	De Paepe et al. (2001)
	qPCR		Increased in carcinomas	Tahmasebi et al. (2006)
	IHC		expressed in both ER+ and ER-	Herr et al. (2008)
	FISH/qPCR		overexpression in 10–20% breast carcinomas	Rhodes et al. (2009)
		MCF7 (subcutaneous)	Candesartan reduced tumor growth	Chen et al. (2013)
		MCF7 (subcutaneous)	Losartan reduced growth of AGTR1-overexpressing tumors	Rhodes et al. (2009)
AT2	qPCR		Increased in carcinomas	Tahmasebi et al. (2006)
	IHC/ISH		Increased in benign and malignant tumors	De Paepe et al. (2002)
MAS	RT-PCR		detected in normal and malignant samples	Miller et al. (1997)
		ZR75-1, BT474 (orthotopic)	Ang1–7 reduces tumor growth	Cook et al. (2010)

*IHC: Immunohistochemistry; ISH: *In situ* hybridization, qPCR: quantitative PCR; FISH: Fluorescent *in situ* hybridization.*

A large-scale meta-analysis performed on 31 breast cancer profiling datasets has revealed over expression of the AT1 receptor gene (AGTR1) in 10–20% of invasive breast tumors (Rhodes et al., 2009), all of which were estrogen receptor positive (ER+) and human epidermal receptor 2 (HER2)-negative. In another subset of 178 HER2-negative breast tumor patients, high AT1 expression was identified as a marker of resistance to anthracyclin-based neoadjuvant chemotherapy (de Ronde et al., 2013). Recent studies further suggested that high AT1 level may be a predictive marker of bevacizumab response in breast tumors (Sánchez-Rovira et al., 2013; Salvador et al., 2014). Of interest, bevacizumab-induced hypertension in breast cancer patients was also associated with better overall response to this anti-angiogenic therapy (Gampenrieder et al., 2014).

### AT2

AT2 receptor levels have been shown to be markedly increased in breast tumors as compared to normal tissue (De Paepe et al., 2002; **Table 1**) raising the question of the functional effect of AT2 over expression in breast cancer. To specifically examine this question, we generated a human metastatic breast cancer cell line stably expressing high amounts of human AT2 receptors at the plasma membrane and no detectable AT1 levels (Rodrigues-Ferreira et al., 2012b). This model allows the characterization of AT2 functions independently of those related to AT1 receptor activation, which is of great interest in the context of AT1 blockade by ARBs. It also offers a unique opportunity to evaluate the consequences of AT2 receptor activation and blockade on breast cancer proliferation, invasion, and migration, as well as on tumor growth and metastasis. This model is also suitable to investigate whether AT2 functions may rely on interaction with intracellular partners (Rodrigues-Ferreira et al., 2015). ATIPs are interesting AT2 partners in the context of cancer, as ATIP1 has been shown to reduce proliferation of pancreatic cancer cells (Seibold et al., 2003) whereas ATIP3, which is down-regulated in breast cancer, reduces tumor growth, and metastasis in experimental mice models (Rodrigues-Ferreira et al., 2009). However, whether the consequence of AT2-ATIPs interaction is to promote or prevent breast cancer progression remains to be elucidated.

### MAS

By quantitative RT-PCR, Harmer et al. (2002) found ACE2 expressed in most human cell lines and tissues examined including breast, indicating that Ang1–7 can be produced in most tissues. Preliminary studies detected Mas receptor at the mRNA level by RT-PCR in both benign and malignant breast tissues (Miller et al., 1997; **Table 1**). These results need to be validated at the protein level but one can speculate that Mas/Ang1–7 axis is present and targetable in breast tumors. Of interest, in orthotopic breast cancer xenograft models, administration of Ang1–7 efficiently reduced tumor volume and weight (Cook et al., 2010) by acting both on tumor cells and microenvironment. Thus, Ang1–7 may be a useful therapeutic component against breast cancer.

## TARGETING ANGIOTENSIN RECEPTORS IN BREAST CANCER

Preclinical studies thus provide a rationale for the potential use of either ARBs or Mas agonist to treat breast cancer patients. To go further, Ang1–7 and ARBs have been assessed in clinics to evaluate pharmacokinetics in phase I/II trial, and association with breast cancer risk, respectively.

### AT1

Eight different ARBs (Losartan, Candesartan, Valsartan, Irbesartan, Telmisartan, Eprosartan, Olmesartan, and Azilsartan; **Figure 1**) are currently approved for the treatment of hypertension (Bader et al., 2012) and thus safely used in patients without major side effect, indicating that these ARBs may be reconsidered for the treatment of others pathologies including breast cancer. Several studies evaluated breast cancer risk in ARBs users. Although one study indicated that ARBs are associated with a modest increased risk of new cancer diagnosis (Sipahi et al., 2010), other groups rather reported that ARBs do not influence the risk of developing a cancer, including breast cancer (Li et al., 2003; Fryzek et al., 2006; ARB Trialists Collaboration, 2011; Azoulay et al., 2012; Bhaskaran et al., 2012; Li et al., 2013). Several studies showed that there is no significant association between the use of ARBs and overall survival of breast cancer patients (Sipahi et al., 2010; Chae et al., 2011; Cardwell et al., 2014). Other analyses indicated that ARBs users with invasive breast cancer have a reduced risk of recurrence (Chae et al., 2011, 2013; Mc Menamin et al., 2012), although in one study ARBs were not associated with breast cancer recurrence (Sørensen et al., 2012), an apparent discrepancy that may reflect variations in AT1 levels among different breast cancer populations examined.

### AT2

Compound C21 is the only non-peptide orally active AT2 receptor agonist tested in several preclinical models with broad potential indication (Unger and Dahlöf, 2010). C21 has been shown to have beneficial effects on blood pressure, cardiac, and kidney functions and inflammation (Steckelings et al., 2011; McCarthy et al., 2013), and its effects in cancer still need to be investigated. Other AT2 ligands are under preclinical development (Bader et al., 2012). In particular the AT2 agonist LP2 compound (Lanthio Pharma) is under evaluation in heart and lung injury (Wagenaar et al., 2013). AT2 antagonists such as PD123319 and its orally active derivative EMA401 were also investigated. Of interest, the highly selective AT2 antagonist EMA401 is under development as a novel neuropathic pain therapeutic agent (Rice et al., 2014). Thus, tools to either block or activate the AT2 receptor (**Figure 1**) are under development but preclinical studies are still needed to elucidate AT2 functions in cancer and to adequately target this receptor, in particular in breast cancer.

### MAS

Targeting Mas receptor is a promising therapeutic option. Ang1–7 was evaluated in phase I clinical study involving eighteen patients with advanced solid tumors from colon, lung, pancreas, prostate, and soft tissues (Petty et al., 2009). Only mild toxicity was observed for high doses of Ang1–7 administrated subcutaneously (Petty et al., 2009). Of interest, in this study Ang1–7 treatment led to clinical benefit for four patients. In a phase I/II clinical study, Ang1–7 was administered before and after chemotherapy in breast cancer patients with no dose-limiting toxicity (Rodgers et al., 2006). These clinical studies suggest that Ang1–7 may be used in patients without major side effect. To go further, new tools to target Mas receptor are under development. These include orally active Ang1–7 derivatives and new Mas agonists such as the peptidic compound CGEN-856 and the non-peptidic AVE-0991 compound (**Figure 1**; Bader et al., 2012; Ferreira et al., 2012). Of note, patents were developed for the use of Ang1–7 as anti-cancer and chemopreventive agents able to prevent and reduce cancer growth, including in breast cancer (Tallant et al., 2011; Tallant and Gallagher, 2014).

## CONCLUSION

Angiotensin GPCRs appear as good therapeutic targets expressed at the cell surface and thus easily targetable with pharmacological drugs or humanized blocking antibodies. ARBs are already widely used to treat hypertension and may be safely used in other therapeutic applications, such as breast cancer, in combination with standard treatments. Targeting angiotensin receptors may be promising in several aspects, since beneficial effects of ARBs and Ang1–7 were observed on both tumor cells and tumor microenvironment (Cook et al., 2010; Chen et al., 2013). ARBs and Ang1–7 were also shown to reduce collagen secretion and deposition (Cook et al., 2010; Diop-Frimpong et al., 2011), enhancing drug delivery and potentiating chemotherapy (Diop-Frimpong et al., 2011; Chauhan et al., 2013). These interesting results open the way to the use of ARBs/Ang1–7 in complement with chemotherapy to improve their efficiency. Of note, protective effects of ARBs in cancer may be potentiated by Mas activation, as suggested in studies of vascular remodeling (Iwai et al., 2012). In addition, ARBs have been shown to reduce cardiotoxicity of anthracyclin-based chemotherapy of breast cancer (Thakur and Witteles, 2014), whereas Ang(1–7) reduces cytopenia of ovarian cancer patients treated with gemcitabine/platinum-based chemotherapy (Pham et al., 2013). Thus using ARBs or Ang1–7 combined with chemotherapy may constitute an interesting strategy to both increase drug efficiency and reduce its side effects.

Future studies should be designed to further evaluate angiotensin receptor levels in breast tumors, in an attempt to select a population of patients that express high levels of AT1 or Mas receptors and may thus benefit from GPCR-targeted therapy.

## Conflict of Interest Statement

The authors declare that the research was conducted in the absence of any commercial or financial relationships that could be construed as a potential conflict of interest.
